# Association Between Surgical Timing and In-Hospital Mortality in Infective Endocarditis: A Retrospective Single-Centre Study

**DOI:** 10.3390/antibiotics15100997

**Published:** 2026-10-09

**Authors:** Adelina Maria Radu, Irina Florentina Talpoși, Raluca Elena Țoțoiu, Roxana Oana Berbecaru, Ana Dobrin, Violeta Melinte, Cristina Maria Văcăroiu, Tiberiu Holban, Matei Cherecheanu Popa, Amalia Călinoiu, Oana Andreea Popa, Mădălina Elena Iancu, Luminița Tomescu, Oana Săndulescu, Valeriu Gheorghiță

**Affiliations:** 1 Faculty of Medicine, Carol Davila University of Medicine and Pharmacy, 050474 Bucharest, Romania; ana.dobrin@drd.umfcd.ro (A.D.); dr.melinte@gmail.com (V.M.); cristinamarv@yahoo.com (C.M.V.);; 2 Agrippa Ionescu Clinical Emergency Hospital, 011356 Bucharest, Romania; irina-florentina.talposi@drd.umfcd.ro (I.F.T.); raluca-elena.totoiu@rez.umfcd.ro (R.E.Ț.); roxana-oana.berbecaru0925@rez.umfcd.ro (R.O.B.); tsh0912@gmail.com (T.H.); acalinoiu@gmail.com (A.C.);; 3 Professor Doctor Matei Balș National Institute for Infectious Diseases, 021105 Bucharest, Romania

**Keywords:** infective endocarditis, surgery, surgical timing, risk assessment, mortality, survival

## Abstract

**Introduction:** Infective endocarditis (IE) remains associated with high mortality, and the optimal timing of surgery in patients without an urgent indication remains uncertain. We evaluated the association between surgical timing and in-hospital mortality. **Methods:** We retrospectively studied 100 adults with definite IE admitted during 2020–2025 at a tertiary care centre in Bucharest, Romania. The ECHO score incorporated aetiology, blood culture time to positivity, vegetation size, and embolisation or surgical indication. Surgical timing was categorised as study-specific descriptive bins: <7 days, 7–14 days, or >14 days after hospital admission. The primary outcome was in-hospital mortality. **Results:** Eighty-two patients had a surgical indication (51 non-urgent and 31 urgent), and 60 underwent surgery. In the pooled indicated cohort, crude mortality was 56.0% at <7 days, 23.1% at 7–14 days, 27.3% at >14 days, and 36.4% with no surgery despite indication (*p* = 0.123). Among patients with a non-urgent indication, mortality was 25.0% (3/12), 25.0% (2/8), 26.7% (4/15), and 25.0% (4/16), respectively, showing no apparent favourable timing interval. Mortality across ECHO score strata 0–1, 2, and 3–4 was non-monotonic (31.8%, 47.6%, and 27.8%); the continuous score showed negligible correlation with mortality (r = −0.05) and an AUC of 0.466 (95% CI 0.353–0.575). **Conclusions:** Crude mortality differed across timing categories when urgent and non-urgent indications were pooled, whereas mortality was similar across timing categories in the non-urgent subgroup. These timing categories are descriptive and cannot identify a favourable or optimal surgical window or support causal treatment comparisons.

## 1. Background

Despite advances in diagnostic and therapeutic strategies, infective endocarditis (IE) remains a serious and potentially life-threatening condition characterized by high morbidity and mortality [1]. Contemporary data report in-hospital mortality rates of 20% to 30%, reflecting the disease’s complexity and frequent complications, such as heart failure, systemic embolization, and uncontrolled infection [2,3].

Early identification of high-risk patients and timely intervention are essential for improving outcomes [4]. Large vegetations and *Staphylococcus aureus* infections have been associated with an increased risk of embolism and worse outcomes [5]. Meanwhile, prosthetic valve- and device-related endocarditis present additional therapeutic challenges [6,7].

Current strategies combine antimicrobial therapy and surgery, with surgery indicated in 50–70% of cases. However, determining the optimal timing of surgery remains challenging [8]. While urgent surgery is recommended in cases of hemodynamic instability, uncontrolled infection, or high embolic risk, the management of non-urgent indications is less clear.

Current ESC 2023 guidelines emphasize that surgical timing in infective endocarditis should be individualized and classified as emergent, urgent, or elective, although the optimal timing in patients with non-urgent indication remains uncertain [9,10].

Previous studies have shown that early surgical intervention in infective endocarditis can reduce embolic events and improve clinical outcomes, particularly in patients with large vegetations or high-risk features, although its impact on overall mortality remains variable [11,12,13].

This study evaluated the association between surgical timing and in-hospital mortality in patients with infective endocarditis, with particular attention to patients with non-urgent surgical indications.

## 2. Results

### 2.1. Recorded Cohort Characteristics

**Figure 1:**
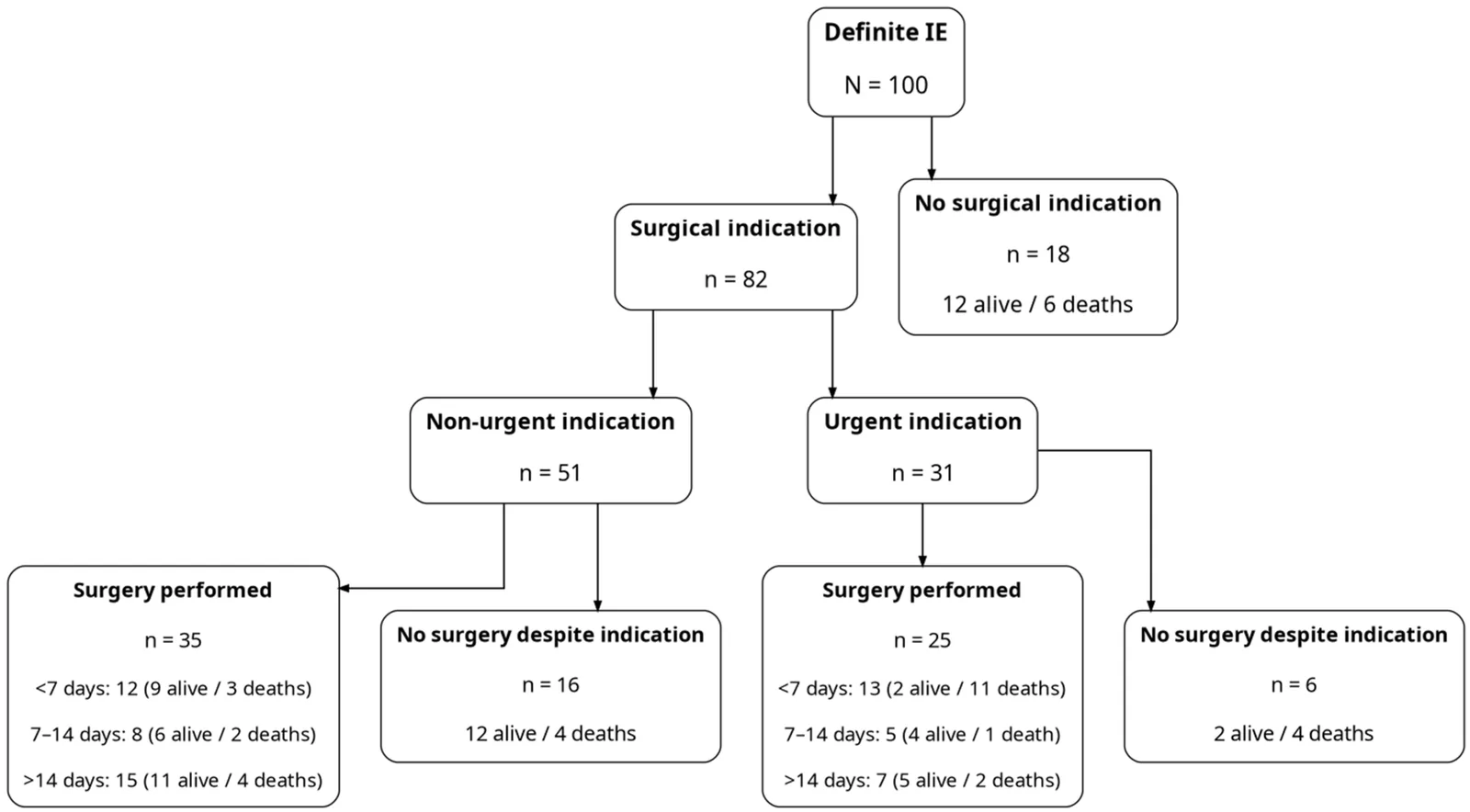
Flow diagram of surgical indication, recorded urgency, operative management, surgical timing, and in-hospital outcome in the study cohort. Of 100 patients with definite infective endocarditis, 82 had a documented surgical indication (51 non-urgent and 31 urgent); 60 underwent surgery and 22 did not undergo surgery despite indication. Surgical timing is shown using the study-specific admission-based categories (<7 days, 7–14 days, and >14 days). Outcomes indicate vital status at hospital discharge.

A total of 100 patients with definite infective endocarditis were included (Figure 1). Median age was 67.5 years (IQR 61.0–73.0), and 72 patients (72.0%) were male. The mean ECHO score was 2.22 ± 0.88; 22 patients (22.0%) had a score of 0–1, 42 (42.0%) had a score of 2, and 36 (36.0%) had a score of 3–4.

*Staphylococcus* spp. were identified in 24.0% of patients and *Candida* spp. in 4.0%; the remaining 72.0% had other aetiologies. Blood cultures became positive within 24 h in 51.0%, vegetations > 10 mm were recorded in 49.0%, and embolic events in 57.0%. These variables were recorded during the index admission and were not uniformly documented as being present at the exact time the surgical indication was established.

A surgical indication was established in 82 patients (82.0%), including 51 (51.0%) with a non-urgent indication and 31 (31.0%) with an urgent indication. Among patients with a surgical indication, 60 (73.2%) underwent surgery: 25 (30.5%) at <7 days, 13 (15.9%) at 7–14 days, and 22 (26.8%) at >14 days after admission. Twenty-two patients (26.8%) did not undergo surgery despite indication. Overall, in-hospital mortality was 37.0%. The database recorded 62 patients as alive at one year; this field is reported descriptively only. Mean length of stay was 39.1 ± 22.7 days (Table 1).

### 2.2. Descriptive Surgical Timing and In-Hospital Mortality

Among all 82 patients with a surgical indication, mortality was 56.0% (14/25; 95% CI 37.1–73.3) in the <7-day group, 23.1% (3/13; 95% CI 8.2–50.3) in the 7–14-day group, 27.3% (6/22; 95% CI 13.2–48.2) in the >14-day group, and 36.4% (8/22; 95% CI 19.7–57.0) among patients who did not undergo surgery despite indication (Table 2).

The unadjusted overall comparison across these future-defined groups was *p* = 0.123. Because the groups combine urgent and non-urgent indications and require survival to the eventual treatment time, these figures are descriptive and are not interpreted as evidence that one surgical interval is preferable.

### 2.3. Exploratory ECHO Composite Index

Mortality was 31.8% (7/22; 95% CI 16.4–52.7) for ECHO scores 0–1, 47.6% (20/42; 95% CI 33.4–62.3) for score 2, and 27.8% (10/36; 95% CI 15.8–44.0) for scores 3–4 (Table 2). The pattern was non-monotonic, and the unadjusted association between score stratum and mortality was not statistically significant (*p* = 0.165). The continuous ECHO score showed negligible correlation with mortality (r = −0.05).

The AUC of the original ECHO score for in-hospital mortality was 0.466 (95% bootstrap CI 0.353–0.575), which is close to chance discrimination and does not support use of the score as a prognostic risk-stratification tool. Mortality did not differ significantly by native-valve, prosthetic-valve, or device-related IE in the original analysis (*p* = 0.89).

### 2.4. Surgical Timing Stratified by Recorded Urgency

Among the 51 patients with a recorded non-urgent surgical indication, mortality was 25.0% (3/12) at <7 days, 25.0% (2/8) at 7–14 days, 26.7% (4/15) at >14 days, and 25.0% (4/16) without surgery despite indication. Thus, the non-urgent population showed no apparent mortality gradient favouring the 7–14-day interval. Among 31 patients with an urgent indication, mortality was 84.6% (11/13), 20.0% (1/5), 28.6% (2/7), and 66.7% (4/6), respectively (Table 3). These urgent-group cells are small and highly selected and are presented only to demonstrate the marked clinical heterogeneity that is obscured by pooling urgency categories.

### 2.5. Patients Who Did Not Undergo Surgery and Secondary One-Year Status

Twenty-two patients had a documented surgical indication but did not undergo surgery. Available clinical documentation showed heterogeneous circumstances, including refusal and deferral, while a specific reason was not consistently recorded for every patient. This group was therefore not treated as a comparable untreated control. The database field indicating 62 patients alive at one year was retained only as secondary descriptive information; no Kaplan–Meier or other post-discharge time-to-event comparison was performed in the revised analysis.

## 3. Discussion

In this retrospective cohort of 100 adults with definite infective endocarditis, crude in-hospital mortality differed across surgical-timing categories when urgent and non-urgent indications were pooled. Within the non-urgent subgroup, mortality was nearly identical across the <7-day, 7–14-day, >14-day and no-surgery categories, at 25.0%, 25.0%, 26.7% and 25.0%, respectively. These findings provide no descriptive support for a preferred or favourable 7–14-day interval in patients with a non-urgent indication. The ECHO composite also failed to demonstrate useful discrimination of in-hospital mortality. Together, these findings limit the use of either the timing categories or the score for directing operative decisions.

Stratification by recorded urgency revealed substantial heterogeneity within the pooled timing groups. Among patients operated on within 7 days, 11 of the 14 deaths occurred in those with an urgent indication. Mortality in this interval was 84.6% in the urgent subgroup and 25.0% in the non-urgent subgroup. This contrast is compatible with confounding by indication, whereby the clinical circumstances prompting earlier surgery are themselves associated with a higher risk of death. Heart failure, uncontrolled infection and embolic complications can influence both operative urgency and prognosis [9]. However, the available data do not establish which factors account for the observed difference or how much of it they explain. The small urgent-subgroup cells and absence of adjustment for clinical severity preclude interpreting the higher early mortality as an adverse effect of early surgery.

Prior observational studies have likewise identified baseline clinical severity and patient characteristics as important predictors of early or in-hospital mortality in infective endocarditis [14,15].

Risk-assessment approaches developed in related *Staphylococcus aureus* bacteraemia populations [16] and multidisciplinary Endocarditis Team models [17] further illustrate the value of structured clinical assessment when treatment decisions are made.

Reviews and meta-analyses comparing early and conventional surgery, including patients with neurological complications, have reported heterogeneous outcomes that depend substantially on case selection, clinical indication, and the definition of surgical timing [18,19,20].

Intravascular catheter-related infection and other infectious complications also contribute to the clinical heterogeneity encountered across infective endocarditis populations [21,22].

Immortal-time bias further restricts interpretation of these comparisons [23]. Membership of a later-surgery category requires survival until the operation, whereas patients who die before reaching that interval cannot enter that group. Defining exposure according to eventual treatment therefore conditions group membership on subsequent survival. Reporting crude proportions and confidence intervals does not remove this bias. Likewise, the non-significant overall comparison (*p* = 0.123) neither establishes equivalence between timing strategies nor excludes a clinically meaningful effect. A model treating surgery as a time-varying exposure could address the allocation of preoperative survival time, but credible causal inference would also require adequate control of factors influencing treatment selection. Such analyses were not supported by sufficiently standardised time-updated severity and operative-risk data in this cohort.

The time origin is also clinically important. Our categories measured the interval from hospital admission to surgery, rather than the interval from establishment of the surgical indication to intervention. For patients with a surgical indication, the recorded diagnosis date and the date of the Endocarditis Team decision establishing the indication were the same recorded time point. However, that time point was not used as the origin for the timing categories analysed here. Consequently, these categories cannot quantify delay after the operative decision. The 2023 ESC Guidelines distinguish emergency surgery within 24 h, urgent surgery within 3–5 days, and non-urgent surgery during the same hospital admission. Our admission-based intervals do not map directly onto those definitions. Moreover, the recorded urgent and non-urgent labels lacked uniform retrospective adjudication against ESC criteria, limiting conclusions about compliance with guideline-defined urgency [9].

The randomised evidence addresses a more specific clinical question. In the EASE trial, Kang and colleagues assigned 76 patients with left-sided infective endocarditis, severe valve disease and large vegetations to surgery within 48 h of randomisation or conventional treatment. Early surgery reduced the composite endpoint of in-hospital death or embolic events, principally through fewer embolic events, without a statistically significant difference in all-cause mortality at 6 months [24]. That trial evaluated a defined treatment strategy from randomisation in a selected population. Its findings cannot be translated directly into an optimal admission-based interval for a heterogeneous cohort containing urgent and non-urgent indications. Conversely, the nearly identical mortality proportions in our non-urgent subgroup do not contradict a benefit of appropriately indicated surgery, because our analysis cannot separate treatment effects from selection and survival biases.

The patients who did not undergo surgery require particular caution. Twenty-two of the 82 patients with an indication remained non-operated, and eight died in hospital. In the prospective ICE-PLUS cohort, surgery was performed in 76% of patients with an indication, illustrating that non-operative management despite indication represents a substantial and clinically selected pathway [25]. In our cohort, documentation included refusal and deferral, but reasons were not consistently available for every patient. We therefore could not distinguish their respective contributions to mortality or compare operated and non-operated patients on an equivalent clinical basis. The 25.0% mortality among non-operated patients with a non-urgent indication should not be interpreted as evidence that withholding surgery is equivalent to intervention.

The ECHO results provide a separate reason for caution in risk stratification. Mortality was non-monotonic across score strata, at 31.8% for scores 0–1, 47.6% for score 2 and 27.8% for scores 3–4. The negligible correlation with mortality (r = −0.05) and AUC of 0.466 (95% bootstrap CI 0.353–0.575) indicate discrimination close to chance in this sample. These findings do not support describing the strata as progressively increasing mortality-risk groups. The lower observed mortality in the highest score stratum also does not establish a protective association. Poor performance of the composite does not negate the clinical relevance of its individual components, but it shows that combining them in this form did not yield a useful mortality predictor.

Several features of the score’s construction may contribute to this performance. Equal weights and binary thresholds were specified without derivation from a multivariable prognostic model. The component assigning one point for embolisation or a surgical indication also overlaps with selection into the timing analysis. By construction, every patient in the indicated cohort receives this point, so the component cannot discriminate between patients within that population. It additionally combines an established complication with a treatment decision, which have different clinical meanings. Furthermore, the component variables were recorded during the index admission and were not uniformly documented at the moment the indication was established. Their temporal relationship to treatment and outcome therefore limits interpretation of ECHO as a baseline prognostic instrument.

The microbiological component warrants further consideration. Santos-Patarroyo and colleagues reported a modest inverse association between blood-culture time to positivity and vegetation length in *Staphylococcus aureus* endocarditis [5]. This observation does not validate a universal 24-h threshold for mortality prediction across all infective endocarditis aetiologies. In our dataset, bottle-level timestamps, handling of multiple positive bottles, preceding antibiotic exposure and consistency of the microbiological platform were not uniformly available for verification. These gaps limit the reproducibility of the component. Assigning the same aetiological weight to all staphylococci and fungi may also obscure differences between organisms. With only four *Candida* cases, this study cannot determine the prognostic contribution of fungal aetiology separately.

The bootstrap confidence interval quantifies uncertainty around the observed AUC, but this calculation does not constitute optimism-corrected internal validation of a developed prediction model. No mapping from ECHO points to individual mortality probabilities was established, and calibration was not assessed. Prognostic model development requires explicit attention to predictor timing, discrimination and calibration, followed by appropriate internal and external validation [26]. The score therefore remains an exploratory composite rather than a clinically validated risk tool. In addition, no formal interaction analysis established whether the association between surgical timing and outcome differed by ECHO stratum. The available evidence consequently does not support score-specific recommendations about operative timing.

The clinical interpretation should remain confined to the outcomes and population studied. Retrospective recruitment at a single centre and sparse urgency-by-timing subgroups limit both precision and generalisability. Similar observed mortality within the non-urgent subgroup cannot establish the safety of deliberate postponement. All-cause in-hospital mortality also does not identify the contribution of individual disease complications or perioperative events. The one-year status field was not analysed as a longitudinal endpoint and provides no basis for comparative claims about longer-term survival. These constraints define the descriptive scope of the findings.

Given the retrospective observational design, limited sample size, small number of events within individual timing categories, and absence of adequate adjustment for time-varying clinical severity, residual confounding remains substantial. Therefore, the lower crude mortality observed in the pooled 7–14-day group should be regarded as a descriptive finding only and should not be interpreted as evidence of a favourable or optimal surgical interval.

Clinically, surgical timing should remain individualised through multidisciplinary Endocarditis Team assessment, guided by guideline-defined urgency and the patient’s evolving clinical status [9]. The findings instead support careful distinction between clinical urgency, elapsed time since admission and elapsed time since the surgical decision when interpreting outcomes and planning further research.

## 4. Methods

### 4.1. Study Design and Setting

We conducted a retrospective observational cohort study at a university-affiliated tertiary care hospital in Bucharest, Romania. The cohort comprised adult patients admitted between January 2020 and December 2025 with definite infective endocarditis. The study evaluated clinical characteristics, microbiology, surgical indication and timing, and in-hospital outcomes.

Reporting followed the Strengthening the Reporting of Observational Studies in Epidemiology (STROBE) framework.

### 4.2. Study Population

The study included 100 adults with definite IE. The cohort was established using the modified Duke criteria as defined by Li et al. [10]. The 2023 Duke-ISCVID criteria were not substituted retrospectively for the original cohort definition because part of the study period preceded their publication.

### 4.3. Data Collection, ECHO Composite, and Surgical Timing

Data were collected retrospectively from hospital records, including demographic characteristics, aetiology, blood-culture time to positivity, vegetation size, embolisation, surgical indication and urgency, timing of surgery, and clinical outcomes. The exploratory ECHO composite assigned one point for each of the following: (E) *Staphylococcus* spp. or fungal aetiology; (C) blood-culture positivity within 24 h; (H) vegetation > 10 mm; and (O) embolisation or a surgical indication. The total score ranged from 0 to 4. The equal weights, component thresholds, and categories 0–1, 2, and 3–4 were study-specific and were not derived from a multivariable prognostic model; these categories are therefore referred to as score strata rather than low-, intermediate-, and high-risk groups.

Blood-culture time to positivity was available in the research dataset as a recorded value/category used for the original ≤24 h component. Bottle-level instrument timestamps, the rule used when several bottles were positive, antibiotic exposure before culture collection, and consistency of the microbiological platform across the entire study period were not available for uniform retrospective verification. The 24 h threshold is therefore study-specific; evidence derived from *Staphylococcus aureus* IE does not establish the same prognostic threshold across all IE aetiologies [5].

Analyses of surgical timing were restricted to the 82 patients with a documented surgical indication. The 51 patients with a recorded non-urgent indication form the principal descriptive population for questions concerning discretionary timing, while the 31 patients with an urgent indication are described separately. Patients with an indication who did not undergo surgery were retained as “no surgery despite indication”; because reasons for non-operation were heterogeneous and incompletely documented, this category was not treated as an exchangeable untreated control group.

For the submitted analysis, surgical timing was calculated from hospital admission to surgery and categorised as <7 days, 7–14 days, or >14 days. These intervals were treated only as descriptive, study-specific bins. For patients with a surgical indication, the recorded date of IE diagnosis and the date on which the Endocarditis Team established the surgical indication represented the same recorded time point. This study-specific operational definition did not necessarily represent symptom onset, the first clinical suspicion of IE, or the first diagnosis at a referring hospital; in transferred patients, the recorded time point could precede admission to our hospital. The timing categories analysed here nevertheless used hospital admission, rather than this recorded diagnosis/indication date, as their time origin. Uniform case-level retrospective adjudication against the 2023 ESC urgency criteria was not available.

The primary outcome was all-cause in-hospital mortality during the index admission. The one-year survival field was retained only as secondary descriptive information and was not analysed as a time-to-event endpoint.

### 4.4. Statistical Analysis

Statistical analysis was performed using standard descriptive and comparative methods. Continuous variables are summarised using mean ± standard deviation (SD) or median with interquartile range (IQR), as appropriate. Categorical variables are presented as frequencies and percentages. Mortality proportions are accompanied by Wilson 95% confidence intervals (CIs).

The association between the continuous ECHO composite index and binary mortality was summarised using the point-biserial correlation coefficient. Discrimination was assessed using the area under the receiver operating characteristic curve (AUC), with a percentile 95% CI obtained from 2000 patient-level bootstrap samples.

Statistical analyses were performed using IBM SPSS Statistics, version 26.0 (IBM Corp., Armonk, NY, USA). Confidence-interval and AUC/bootstrapping calculations were verified using Python, version 3.10 (Python Software Foundation, Wilmington, DE, USA). A two-sided *p*-value < 0.05 was considered statistically significant for formal comparisons.

### 4.5. Ethics

The study was approved by the Clinical Emergency Hospital Prof. Dr. Agrippa Ionescu Ethics Committee. Informed consent was waived due to the study’s retrospective nature. All data were anonymised and analysed according to the Declaration of Helsinki.

## 5. Study Limitations

This study has several important limitations. It is retrospective, single-centre, and includes only 100 patients, with small numbers in the urgency-by-timing subgroups. Surgical timing was originally grouped from hospital admission rather than from the date the indication was established, so these categories cannot measure delay from surgical decision. Recorded urgent/non-urgent labels were not supported by a uniform case-level retrospective adjudication against 2023 ESC criteria. The eventual timing groups are vulnerable to immortal-time bias, and the available dataset did not contain sufficiently standardised time-updated severity and operative-risk variables for a credible causal time-dependent treatment model. Reasons for non-operation were incompletely documented. The exploratory ECHO composite was ad hoc, contains a component that overlaps with surgical selection, and did not demonstrate useful discrimination. Detailed microbiological time-to-positivity procedures were not uniformly available, and one-year status was not analysed as a longitudinal endpoint.

## 6. Future Directions

Additional prospective, multicentre studies are necessary to validate these findings and better define optimal timing strategies for infective endocarditis. Combining dynamic clinical variables and advanced risk models may improve patient stratification and guide therapeutic decisions.

## 7. Conclusions

In this retrospective cohort, crude mortality differed across post-admission timing categories when urgent and non-urgent indications were pooled; however, these differences should not be interpreted as evidence favouring any surgical interval. The non-urgent subgroup showed essentially identical mortality across timing categories. The ECHO score showed non-monotonic mortality across strata and an AUC close to chance; it should therefore remain an exploratory composite and should not guide clinical decisions.

The study does not provide sufficient evidence to identify a favourable or optimal surgical interval. Surgical timing in infective endocarditis should therefore be individualised by a multidisciplinary Endocarditis Team, guided by guideline-defined urgency and the patient’s evolving clinical status.

## Figures and Tables

**Table 1:** Recorded Clinical, Microbiological, Echocardiographic, and Outcome Characteristics.

Characteristic	Value
Number of patients	100
Age, median (IQR), years	67.5 (61.0–73.0)
Male sex	72 (72.0%)
ECHO score (mean ± SD)	2.22 ± 0.88
ECHO score 0–1	22 (22.0%)
ECHO score 2	42 (42.0%)
ECHO score 3–4	36 (36.0%)
*Staphylococcus* spp.	24 (24.0%)
*Candida* spp.	4 (4.0%)
Other aetiology	72 (72.0%)
Blood culture ≤ 24 h	51 (51.0%)
Vegetation > 10 mm	49 (49.0%)
Embolisation	57 (57.0%)
Any surgical indication	82 (82.0%)
Non-urgent indication	51 (51.0%)
Urgent indication	31 (31.0%)
No indication	18 (18.0%)
Surgery performed (among indicated)	60 (73.2%)
<7 days (among indicated)	25 (30.5%)
7–14 days (among indicated)	13 (15.9%)
>14 days (among indicated)	22 (26.8%)
No surgery despite indication	22 (26.8%)
Alive at hospital discharge	63 (63.0%)
Death	37 (37.0%)
Relapse	4 (4.0%)
1-year survival	62 (62.0%)
Length of stay, mean ± SD, days	39.1 ± 22.7

Values are n (%) unless otherwise stated. Age and sex refer to admission. Other clinical, microbiological, imaging, and treatment variables were recorded during the index hospitalisation and were not uniformly available at one common baseline time point. The submitted surgical-timing categories are measured from hospital admission and are descriptive only.

**Table 2:** Descriptive In-Hospital Mortality According to Submitted Surgical-Timing Categories and ECHO Score Strata.

Timing/ECHO Score Stratum	Alive	Death	Mortality %	95% CI
<7 days	11	14	56.0%	37.1–73.3
7–14 days	10	3	23.1%	8.2–50.3
>14 days	16	6	27.3%	13.2–48.2
No surgery despite indication	14	8	36.4%	19.7–57.0
**ECHO Score Stratum (Overall Cohort)**	**Alive**	**Death**	**Mortality %**	**95% CI**
0–1	15	7	31.8%	16.4–52.7
2	22	20	47.6%	33.4–62.3
3–4	26	10	27.8%	15.8–44.0

Mortality percentages and Wilson 95% CIs are calculated within each timing or ECHO score stratum. Timing categories are defined from hospital admission and should not be interpreted as causal treatment groups. *p* = 0.123 for the unadjusted timing-category comparison; *p* = 0.165 for the unadjusted ECHO-strata comparison.

**Table 3:** Descriptive In-Hospital Mortality by Submitted Timing Category and Recorded Surgical Urgency.

Recorded Urgency/Timing	Patients	Deaths	Mortality %	95% CI
Non-urgent: <7 days	12	3	25.0%	8.9–53.2
Non-urgent: 7–14 days	8	2	25.0%	7.1–59.1
Non-urgent: >14 days	15	4	26.7%	10.9–52.0
Non-urgent: no surgery	16	4	25.0%	10.2–49.5
Urgent: <7 days	13	11	84.6%	57.8–95.7
Urgent: 7–14 days	5	1	20.0%	3.6–62.4
Urgent: >14 days	7	2	28.6%	8.2–64.1
Urgent: no surgery	6	4	66.7%	30.0–90.3

Wilson 95% CIs are shown. The categories are defined by eventual timing from admission and are descriptive only; no causal or optimal-timing inference is made from these sparse subgroups.

## Data Availability

The data presented in this study are not publicly available due to patient privacy and ethical restrictions. De-identified data may be made available from the corresponding author upon reasonable request and with permission from the Institutional Ethics Committee, in accordance with institutional policies and applicable data protection regulation.
